# Roles of helminth extracellular vesicle-derived let-7 in host–parasite crosstalk

**DOI:** 10.3389/fimmu.2024.1449495

**Published:** 2024-10-28

**Authors:** Haoran Zhong, Guiquan Guan, Yamei Jin

**Affiliations:** ^1^ National Reference Laboratory for Animal Schistosomiasis, Shanghai Veterinary Research Institute, Chinese Academy of Agricultural Sciences, Shanghai, China; ^2^ Key Laboratory of Animal Parasitology of Ministry of Agriculture and Rural Affairs, Shanghai Veterinary Research Institute, Chinese Academy of Agricultural Sciences, Shanghai, China; ^3^ State Key Laboratory for Animal Disease Control and Prevention, Lanzhou Veterinary Research Institute, Chinese Academy of Agricultural Science, Lanzhou, Gansu, China; ^4^ Key Laboratory of Veterinary Parasitology of Gansu Province, Lanzhou Veterinary Research Institute, Chinese Academy of Agricultural Science, Lanzhou, Gansu, China

**Keywords:** helminth, extracellular vesicles, microRNA, Let-7 family, host-parasite interaction

## Abstract

Helminth infections are a major public health problem as they can cause long-term chronic infections in their hosts for which there is no effective vaccine. During the long-term interaction between helminths and their hosts, helminth-derived extracellular vesicles (EVs) can participate in host immunomodulatory processes by secreting bioactive molecules (BMAs). Growing data suggests that microRNAs (miRNAs) in helminth EVs have a significant impact on the host’s immune system. The let-7 family is highly conserved among helminth EVs and highly homologous in the host, and its function in host–parasite crosstalk may reflect active selection for compatibility with the host miRNA machinery. In-depth studies targeting this aspect may better elucidate the mechanism of parasite-host interactions. Hence, this review summarizes the current studies on the cross-species involvement of helminth EV-derived let-7 in host immune regulation and discusses the barriers to related research and potential applications of helminth EVs.

## Introduction

Helminthiosis, caused by nematodes, cestodes, and trematodes, are a major public health concern and result in ∼60 million disability-adjusted life years (DALYs) globally (1). Recent estimates indicate that 2 billion people in low- and middle-income countries, mostly in endemic areas of Asia, the Americas, and Africa, have been infected by one or more parasitic worms (2). Unfortunately, there are currently no broad-spectrum anthelmintic vaccines available (3). Therefore, most helminth infections are controlled with the use of anthelmintic drugs, which do not prevent reinfection (4). However, prolonged and over use of these drugs can lead to the development of anthelmintic resistance (5–7). Hence, new control and prevention strategies against helminth infections are urgently needed.

As a result of complex life cycles and long-term symbiosis with the host, helminths release various mediators involved in host–parasite crosstalk, which promote immunomodulation, immune evasion, and pathogenesis (8, 9). Previous studies have primarily focused on helminth excretory/secretory products (ESPs) involved in regulating the host immune response (10, 11). However, as the study of host–parasite interactions has entered a deeper molecular realm, there has been a gradual emergence of helminth-derived microRNAs (miRNAs), mainly loaded in extracellular vesicles (EVs), involved in this intricate crosstalk (9, 12, 13).

EVs are small membrane-bounded secretory vesicles released by almost all cell types. EVs play essential roles in cell–cell communication via a wide variety of bio-active molecules (BMAs), including small RNAs, DNAs, messenger RNAs (mRNAs), proteins, lipids, and glycans (14, 15). These BMAs are encapsulated by EVs that are protected by a membrane to prevent degradation in the extracellular environment (16). Among these diverse BMAs conveyed by EVs, miRNAs are regarded as multifunctional regulators of various biological functions (17). MiRNAs play a pivotal role in regulating gene expression, affecting critical cellular functions such as growth, differentiation, and apoptosis (18). Their biogenesis begins with the transcription of primary miRNA (pri-miRNA) by RNA polymerase II. This pri-miRNA is processed in the nucleus by the Drosha-DGCR8 complex into precursor miRNA (pre-miRNA), which is then transported to the cytoplasm via Exportin-5. In the cytoplasm, Dicer further processes the pre-miRNA into mature miRNA. The mature miRNA is then integrated into the RNA-induced silencing complex (RISC), where it directs the repression of target mRNAs through sequence-specific binding, thereby modulating various biological processes and contributing to disease mechanisms (19). However, little is known about the mechanisms that drive and regulate the incorporation of miRNAs into helminth EVs and how specific miRNAs are preferentially selected as cargo (20). Nonetheless, both helminth EV-derived miRNAs and corresponding host targets are remarkably similar, suggesting that the packaging and release mechanisms of specific miRNAs during long-term host–parasite interactions and related immunomodulatory functions are evolutionarily conserved (13).

The most common miRNA clusters in helminth EVs are let-7, miR-2, miR-9, miR-10, miR-31, miR-71, miR-87, and miR-125 (21). As a conserved miRNA family that is highly homologous to the host, the function of helminth EV-derived let-7 in host–parasite interactions may reflect active selection for compatibility with the host miRNA machinery. This is because the let-7 miRNA from helminths and hosts may function similarly due to their similar sequences, and that the parasite miRNA, once taken up by the host cell, may rely on the host miRNA machinery to modulate gene expression (13). As one of the earliest identified miRNAs, let-7 is associated with reproductive development of *Caenorhabditis elegans*, while deficiency or overexpression of let-7 can lead to developmental abnormalities (22, 23). Although the importance in reproductive development of helminths remains unclear, let-7 can regulate host immune responses through EVs (24–27). Target gene prediction software is often used to analyze host target genes against the let-7 family-specific seed sequence (GAGGUAG) or the full-length sequence of helminth let-7 (26–29). Most of these target genes are associated with the TGF-β, MAPK, and Wnt signaling pathways, which play crucial roles not only in cell proliferation and differentiation but also in host immune and inflammatory reactions. This multiple involvement suggests different roles of helminth EV-derived let-7 in host–parasite crosstalk (13). Therefore, an in-depth study of helminth EV-derived let-7 will further enrich our understanding of this crosstalk and may further clarify the mechanisms underlying the crosstalk between helminth EVs and host cells.

Therefore, the aim of this article is to summarize current studies on the cross-species involvement of helminth EV-derived let-7 in host immune regulation (**Table 1**), while addressing shortcomings of related research and potential applications of helminth EVs.

**Table 1:** Current research involving helminth EV-derived let-7 and their target genes.

Category	miRNA name	Species	EV Origin	EV Purification method	Target gene	Reference
**Trematodes**	sja-let-7	*Schistosoma japonicum*	Adult worm ESPs	Dialysis, ultrafiltration, and ultracentrifugation combined with commercial kit	COL1A2	(24)
	sma-let-7	*Schistosoma mansoni*	Adult worm ESPs	Differential centrifugation followed by membrane filtration and sucrose density ultracentrifugation	COL1A2, MAP3K1, MSN, RASGRP1, SMAD2, FBXO32, LTN1, MUC20, RANBP2, RICTOR	(21)
	fhe-let-7	*Fasciola hepatica*	Adult worm ESPs	Ultracentrifugation	CD200R1, CNOT4, HIF1AN, HOXA9, HSPA14, MAP3K1, MAPK8, PMAIP1, PTAFR, SMAD4, SNAP23, TNFRSF1B, TP53, TRIM71, ZBTB16	(30)
	csi-let-7	*Clonorchis sinensis*	Adult worm ESPs	Ultracentrifugation, iodixanol-based density gradient centrifugation	SOCS1, CLEC7A	(26)
	dde-let-7	*Dicrocoelium dendriticum*	Adult worm ESPs	Ultracentrifugation coupled to membrane filtration	Not validated	(52)
	tsp-let-7	*Trichinella Spiralis*	Larvae ESPs	Ultracentrifugation	Not validated	(53)
**Cestodes**	tpi-let-7	*Taenia pisiformis*	Larvae ESPs	Ultracentrifugation	C/EBP δ, NFκB2	(25, 27)
	egr-let-7	*Echinococcus granulosus*	Protoscolece ESPs Hydatid fluid	Ultracentrifugation	IGF2R	(43)
**Nematodes**	hpo-let-7	*Heligmosomoides polygyrus*	Adult worm ESPs	Ultracentrifugation	DUSP1	(16)
	bma-let-7	*Brugia malayi*	Adult worm ESPs	Commercial kit	EIF4E	(50)
	asu-let-7	*Ascaris suum*	Adult worm, L3, L4 larvae ESPs Adult worm body fluid	Ultracentrifugation, size exclusion chromatography	CD86, PARP8, SENP1, TRIM32 USP15	(49)

EVs, Extracellular vesicles; ESPs, Excretory/secretory products.

## Helminth EV-derived let-7 in host-parasite crosstalk

### Trematodes

Diseases caused by blood fluke (e.g. schistosomiasis), liver flukes (clonorchiosis, opisthorchiosis, fascioliosis etc.) and lung flukes (paragonimiosis) severely hamper health and productivity of humans and animals (24, 26, 30, 31). These diseases induce severe pathological impacts, including chronic inflammation, fibrosis, and extensive tissue damage in affected organs. Specifically, schistosomiasis and fascioliosis primarily target the liver, clonorchiosis and opisthorchiosis lead to biliary obstruction and elevate the risk of cholangiocarcinoma, while paragonimiosis causes pulmonary fibrosis and systemic symptoms due to parasite migration (32, 33).

**Figure 1:**
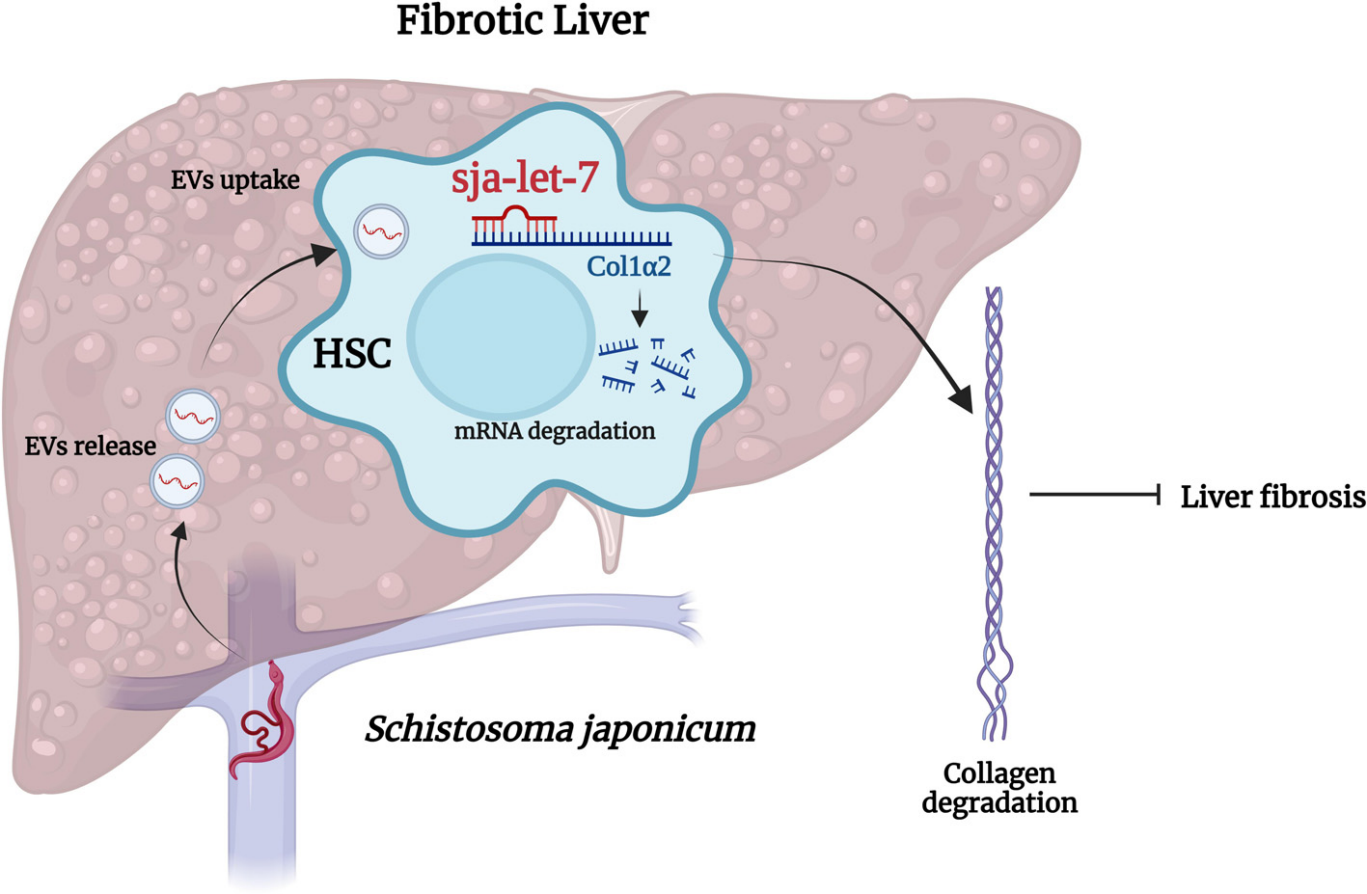
Cross-species sja-let-7 mediates host–parasite interactions in schistosome-induced liver fibrosis. *Schistosoma japonicum* worms residing in the host portal vein release *Sj*EVs that contain sja-let-7, which alleviates schistosome-induced liver fibrosis through the regulation of Col1α2. This figure was created with Biorender.com.

Schistosomes are dioecious, presenting as separate male and female sexes (34). Following dialysis, ultrafiltration, and ultracentrifugation combined with a commercial kit, EVs derived from male and female *S. japonicum* were successfully enriched from the supernatant of *in vitro* cultures and contained a high abundance of sja-let-7 (24, 31, 35). Our group recently reported that *Sj*EVs reduced expression of collagen type I alpha 2 chain (Col1α2), one of the host target genes of sja-let-7, thereby alleviating liver fibrosis (24). The same relationship between helminth let-7 and host Col1α2 was also reported in *S. mansoni* (21). Since sja-let-7 is not highly abundant in *S. japonicum* egg ESPs or EVs (36, 37), this regulatory effect might not be caused by eggs deposited in the host liver, but rather remotely mediated via worm-derived EVs, suggesting that worms can manipulate the host immune responses to alleviate pathological damage, thereby preventing expulsion and promoting a state of tolerance that benefits the parasite by prolonging the host’s life (12). Therefore, it is not surprising that helminth miRNAs can inhibit the pathological processes underlying the establishment of chronic infection (**Figure 1**). However, due to the large number of mRNAs targeted by miRNAs, the consequent biological functions may vary at different stages of infection (18). Fontenla et al. (30) reported that EVs from *F. hepatica* contained fhe-let-7 that could potentially interact with target genes related to different regulatory roles, although the specific mechanisms remain unclear. Notably, let-7 of *C. sinensis* promotes inflammation in the host (26). Yan et al. (26) isolated EVs from *C. sinensis* (*Cs*EVs) by ultracentrifugation combined with iodixanol-based density gradient centrifugation. Further analysis revealed that csi-let-7a, a highly enriched miRNA delivered by *Cs*EVs, plays a pivotal role in the activation of M1-like macrophages and subsequent proinflammatory responses to biliary injury by targeting the host genes suppressor of cytokine signaling 1 and c-type lectin domain containing 7A (26).

### Cestodes

Cestodes are zoonotic parasites that are usually widely spread in susceptible impoverished areas with poor hygiene practices, especially urban settings with livestock (38). In taenid cestodes such as *Taenia solium*, *T. saginata*, and *Echinococcus* spp., an intermediate host ingests contaminated vegetation or food containing cestode eggs, which hatch into larvae in the intestines. These larvae are then transported through the bloodstream to various tissues where they develop into cysts or metacestodes. When the definitive host consumes the infected meat, the infection is established. These metacestodes can cause chronic pathology, reflecting the parasite’s evolved mechanisms to evade the host’s immune response. In humans, *T. solium* can lead to cysticercosis, causing severe neurological damage, while *Echinococcus* spp. may result in hydatid disease with life-threatening cysts in the liver and lungs. In livestock, these infections cause economic losses due to reduced productivity, meat condemnation, and increased veterinary costs (39).

*Cysticercus pisiformis*, the larval stage of *Taenia pisiformis*, can infect rabbits and cause digestive disorders and growth retardation, resulting in great economic losses to the breeding industry. Co-incubation of RAW264.7 macrophages with EVs of *T. pisiformis* (*Tp*EVs) collected by ultracentrifugation increased levels of tpi-let-7 in the cell, which reduced M1 phenotype expression and enhanced M2 phenotype polarization by inhibiting the target gene transcription factor CCAAT/enhancer-binding protein δ (27, 40). Likewise, co-incubation of rabbit peritoneal macrophages with *Tp*EVs resulted in detectable levels of tpi-let-7 in the host cells, demonstrating the delivery of tpi-let-7 by *Tp*EVs (25). Moreover, another potential target gene of tpi-let-7, nuclear factor kappa B subunit 2, was differentially expressed, as determined by proteomic analysis of the same cell samples, suggesting that the tpi-let-7/NFκB2 axis may also be involved in modulating the host immune response by *Tp*EVs (25).

Echinococcosis is a zoonotic disease induced by adult or larval cestodes of the genus *Echinococcus* (41). *Echinococcus granulosus* is a medically significant cestode and a public health concern (42). EVs of *E. granulosus* collected by ultracentrifugation contained high levels of egr-let-7, which might play an immunomodulatory role by targeting insulin like growth factor 2 receptor, although the specific mechanisms remain unclear (43).

### Nematodes

Nematodes are highly diverse, widely distributed, and well-adapted to almost all habitats. Based on complex and varied evolutionary histories, nematodes can be categorized into three main groups: free-living, saprophytic, and parasitic (44).

*Heligmosomoides polygyrus* is a gastrointestinal nematode that infects mice and belongs to the same nematode clade as *C. elegans* (45). During long-term interactions with the host, *H. polygyrus* may induce a strong Th2 response and simultaneously secrete immunomodulatory molecules, which can suppress the host immune response (46). EVs isolated from *H. polygyrus* (*Hp*EVs) by ultracentrifugation were reported to suppress type 2 innate responses and reduce eosinophilia in mice (16). Subsequent microarray analysis revealed that hpo-let-7 from *Hp*EVs could suppress expression of dual specificity phosphatase 1, which is a crucial regulator of inflammation and immunity, to manipulate host cells. In addition to *H. polygyrus*, *Haemonchus contortus* is another significant gastrointestinal nematode that affects livestock, particularly small ruminants such as sheep and goats (47). *H. contortus* is a major cause of parasitic gastroenteritis, leading to severe anemia, reduced productivity, and significant economic losses in the livestock industry (48). Although the specific role of let-7 in *Hc*EVs remains underexplored, understanding its involvement in parasite-host interactions could open new avenues for developing targeted therapies and diagnostic tools for managing infections in livestock.

Interactions between helminth EV-derived let-7 and host targets have also been reported in other nematodes (49, 50). *Brugia malayi* is a pathogen of lymphatic filariasis, commonly known as elephantiasis, a neglected tropical disease that affects millions of people, and an important public health issue due to the high prevalence. Ricciardi et al. (50) isolated EVs from *B. malayi* with a commercial kit that were absorbed by human dendritic cells and monocytes, which downregulated the mammalian target of rapamycin pathway in host cells via the bma-let-7/eukaryotic translation initiation factor 4E axis. *Ascaris suum* is a prevalent parasitic nematode that infects pigs and was found to reduce nutrient utilization and weight gain, ultimately resulting in production loss (51). Through ultracentrifugation and size exclusion chromatography (SEC), EVs were obtained from *A. suum* adults, as well as L3 and L4 larvae, for miRNA profiling (49). The results of target gene prediction showed that asu-let-7 interacted with CD86, which facilitates T-cell activation, suggesting that this axis might be involved in host–parasite interactions (49).

In addition to these species, let-7 has been identified in the EVs of other helminths, although the specific target genes have not yet been identified (52, 53). Details can be found in **Table 1**.

## Current challenges and future perspectives

Although current evidence confirms that the helminth EV-derived let-7 cluster is involved in various pathological and physiological processes, considerable challenges still exist to gain a deeper understanding of the roles of let-7 in host–parasite crosstalk.

First, helminth EVs are typically isolated from *in vitro* culture supernatants of a single formulation, such as Dulbecco’s modified Eagle’s medium or Roswell Park Memorial Institute 1640 medium supplemented with EV-free fetal bovine serum and antibiotics (24–26, 52–55). However, the viability of the worms is gradually decreased in these mediums, which may compromise the quality of helminth EVs. In addition, the evolutionary history of helminths is complex and the acquisition of essential substances from the host varies among parasitized stages. Whether there are differences between helminth EVs collected by *in vitro* culture and those involved in host–parasite interactions remain unknown. Recent studies have reported medium suitable for the reproductive development of some helminths *in vitro* (56, 57). Thus, the use of such a medium to obtain helminth EVs may be a direction for further research.

Second, in most studies, helminth EVs are collected by ultracentrifugation of ESPs as the primary separation and isolation method (25–27, 37, 39, 40, 53–55, 58). However, there has been a recent shift towards the use of other methods, such as SEC (59). While ultracentrifugation is commonly used to isolate EVs, it can compromise vesicle integrity due to high shear forces (60). SEC offers an alternative by separating molecules based on size through a column of porous beads, which enables high-purity isolation of EVs, preserving their biological activity and ensuring reproducibility. Additionally, SEC is scalable for larger volumes, making it ideal for studies requiring substantial quantities of purified EVs (61). Since the collection method influences the number, purity and type of enriched helminth EVs, each study should include detailed information based on a recent publication, titled “Special considerations for studies of extracellular vesicles from parasitic helminths: A community-led roadmap to increase rigor and reproducibility” (62).

Third, further studies are needed to determine whether helminth miRNAs are delivered to host cells as primary or precursor miRNAs, how miRNAs are processed and loaded in the EVs, and whether there are differences in the predicted target genes *in vivo* vs. *in vitro*. Investigating these mechanisms is crucial for advancing our understanding of host-parasite interactions. Future research should focus on exploring the molecular machinery involved in miRNA incorporation into EVs and identifying factors that determine the selectivity of miRNA cargo. These studies will provide valuable insights into how helminth EVs modulate host immune responses and contribute to parasite survival. To preliminarily confirm the interactions *in vitro*, our group conducted fluorescence *in situ* hybridization to label co-localized sja-let-7 and the related target gene Col1α2 in liver sections of mice with schistosome-induced liver fibrosis (24).

Fourth, to explore the biological functions of helminth EVs or miRNAs, recent studies have applied mimics either directly to a single cell type or directly to mice via tail vein injection. However, the use of mimics differs from natural helminth infection. Also, since miRNAs target a large number of mRNAs, potential side effects in the host should be considered. Interestingly, our group administered a mimic of sja-let-7, which resulted in a relatively high abundance miRNAs in *Sj*EVs and an anti-fibrotic effect in mice with schistosome-induced liver fibrosis, as well as carbon tetrachloride-induced liver fibrosis, which alleviated pathological changes, demonstrating the potential application of helminth-derived miRNAs (63). Certainly, it is also necessary to assess safety in future studies.

## Conclusions

This article summarized the roles of helminth EV-derived let-7 in host–parasite interactions. In addition to helminth let-7, in-depth studies are warranted to clarify the biological functions of other BMAs, such as miR-71 (21, 37) and miR-125 (43, 64), to further elucidate potential roles on host–parasite crosstalk and provide a theoretical basis for helminth prevention and control. Moreover, the potential therapeutic and diagnostic applications of let-7 are promising areas for future research. As understanding of the molecular mechanisms governing let-7 and its role in modulating host immune responses deepens, targeted therapies could be developed to inhibit or enhance specific pathways affected by let-7. Similarly, the presence of let-7 in extracellular vesicles could serve as a biomarker for parasitic infections, aiding in the early diagnosis and monitoring of disease progression.
